# A Validation Study of the Work Need Satisfaction Scale for Korean Working Adults

**DOI:** 10.3389/fpsyg.2021.611464

**Published:** 2021-06-24

**Authors:** Ji-Hye Kim, Kieun Yoo, Seran Lee, Ki-Hak Lee

**Affiliations:** Department of Psychology, Yonsei University, Seoul, South Korea

**Keywords:** need satisfaction, scale validation, factor analyis, working adults, psychology of working theory

## Abstract

This study aimed to verify the reliability and validity of the Korean version of the Work Need Satisfaction Scale for working adults, based on the psychology of working theory. A total of 589 working adults in Korea responded to the online survey. Of these respondents, 339 were used for exploratory factor analysis and 250 for confirmatory factor analysis. In Stage 1, we translated all items into Korean, back-translated them into English, and then verified the accuracy of the translation. Exploratory factor analysis revealed the 5-factor structure of the Korean version of the Work Need Satisfaction Scale reflecting those of the original scale (survival needs, social contribution needs, autonomy, relatedness, and competence). The scale showed good internal consistency. In Stage 2, confirmatory factor analysis was conducted, and the results indicated that there were no significant differences between a correlational model, a higher-order model, and a higher-order self-determination needs model. Thus, we offered a higher-order self-determination needs model, which had better model fit and was consistent with the original scale and the psychology of working theoretical framework. In addition, convergent and discriminant validity were supported by correlation estimates of the Korean version of the Work Need Satisfaction Scale, and the concurrent validity showed that the Korean version of the Work Need Satisfaction Scale had a significant proportion of explained variance for outcomes. The findings support the conclusion that this study established strong internal consistency and validity for the Korean version of the Work Need Satisfaction Scale. Thus, the scale is unique and meaningful for measuring need satisfaction in work settings in Korea.

## Introduction

Decent work, as put forth by the Psychology of Working Theory (PWT; Duffy et al., 2016), is an aspirational goal for satisfying three fundamental needs: survival, connection, and self-determination. Once decent work is achieved, people experience work fulfillment and well-being (Duffy et al., 2016). Decent work can accordingly be viewed as an important means for the survival of individuals in society, for connecting with others, and for achieving self-determination. The PWT assumes that one important function of decent work is the satisfaction of primary human needs.

As people spend the majority of their active time at work, work is an important aspect of their lives (Near et al., 1980). As a specific domain of human behavior, work can be influenced by a variety of psychological mechanisms that cause either satisfaction or dissatisfaction. Given that dissatisfaction arises when needs are not met, it is necessary to determine whether those needs have not been fulfilled through work (Schaffer, 1953). Furthermore, need satisfaction in the work setting has a spillover effect on satisfaction in other areas of life (Sirgy et al., 2001). Previous studies have demonstrated the positive influence of need fulfillment on work-related variables (e.g., Gagné and Deci, 2005; Lynch et al., 2005; Van den Broeck et al., 2008) and individual well-being or mental health (e.g., Baard et al., 2004; Lucas et al., 2004; Diener et al., 2015). Thus, fulfillment of work-related needs is essential for working people.

South Korea ranks third among Organisation for Economic Co-Operation and Development (OECD) countries in work-hour duration; thus, need fulfillment at work is likely to have a strong influence on quality of life among Korean working adults. However, few studies provide empirical support for this hypothesis. Currently, Korea is witnessing changes in people's work-related perceptions with an increasing number of voices calling for the enhancement of work conditions, such as decreased working hours (Korea Statistics, 2019b), guaranteed free time and rest time (Nam and Kim, 2019), and policies for addressing workplace bullying (Kim G. J., 2019). Thus, need fulfillment at work has become a critical issue that is drawing serious attention. The primary obstacle preventing further research on this topic is the lack of an appropriate scale. Therefore, this study aimed to adapt and validate the PWT-based Work Need Satisfaction Scale (WNSS; Autin et al., 2019) for use among Korean working adults. Our work will contribute to understanding the importance of needs fulfillment at work and to identifying the effect of needs on the well-being of individuals and organizations for Korean workers under highly demanding conditions. This study is an extension of recent research validating the Decent Work Scale (DWS) in Korean working adults (Nam and Kim, 2019). In this context, the study aimed to adapt and validate the scale to measure need satisfaction at work which helps to broaden current understandings of work experiences among Koreans.

## Theoretical Background

Previous psychological theories related to work placed importance on internal factors such as interest and aptitude but did not prioritize the consideration of people with external barriers and limited options (Blustein, 2008). To compensate for these limitations, the PWT can serve as a framework for understanding broad work experiences, including those of marginalized groups with highly restricted freedom of career choice. Furthermore, it is increasingly important to understand the effects of economic, social, and cultural factors on work, as the labor market is experiencing rapid changes caused by internationalization, increases in unemployment and temporary employment, and technological advancements (Blustein, 2013; Brynjolfsson and McAfee, 2014). The PWT attaches considerable importance to the effects of external constraints on career development (Duffy et al., 2016).

The core concept of the PWT is decent work. Duffy et al. (2016) defined and conceptualized decent work on the basis of standards developed by the International Labor Organization (2008, 2013). Specifically, decent work consists of the following dimensions: “(a) physically and interpersonally safe working conditions (e.g., the absence of physical, mental, and emotional abuse), (b) access to adequate health care, (c) adequate compensation, (d) hours that allow for free time and adequate rest, and (e) organizational values that complement family and social values” (Duffy et al., 2016, p. 130). Predictors that affect decent work include economic constraints and marginalization. These external factors impede the formation of psychological resources such as work volition and career adaptability (Duffy et al., 2016). The PWT further posits that securing decent work will have a positive effect on personal fulfillment and well-being through the satisfaction of needs related to survival, social connectedness, and self-determination (Duffy et al., 2016).

As most of the studies published after the development of the PWT have focused on verifying the predictors of decent work, few studies have investigated the effect of decent work on various outcomes. Therefore, to bridge this gap in the literature, the WNSS was developed (Autin et al., 2019). While the PWT has been intensely studied abroad, it has only recently become a topic of interest in Korea. Reports available from Korea verified that structural constraints (social class and gender discrimination) negatively affected working people, women, and college students (Ahn, 2019; Kim et al., 2019; Lee and Lee, 2020). Additionally, a study validated the DWS for use in Korean working adults (Nam and Kim, 2019). This preliminary research suggests that despite being developed in the context of individualistic cultures, the PWT can also be applied to other cultures (Duffy et al., 2016). However, due to the lack of an appropriate scale for measuring work need satisfaction, we have not been able to link decent work and well-being. Need satisfaction scales developed in South Korea are mostly based on the self-determination theory (SDT) and usually target students (e.g., Jeong and Shin, 2010; Kim A. Y., 2010; Lim and Kim, 2015). Consequently, they fail to offer comprehensive explanations of physiological and social needs (Kim D., 2019) and are not suitable for identifying need satisfaction in work settings. Domain-specific measures have been theorized to be strongly related to domain-specific indicators of optimal functioning (Vallerand, 1997; Van den Broeck et al., 2010). Moreover, a work need satisfaction scale for the Korean context is necessary, given the severity of social structural problems in the Korean labor market such as employment instability and temporary employment. Therefore, in this study, we aimed to validate the PWT-based WNSS for the Korean population.

### Need Satisfaction

PWT studies present survival, social connectedness, and self-determination (autonomy, relatedness, competence) as three basic needs that can be fulfilled through work (Blustein et al., 2008; Duffy et al., 2016). However, work can also negatively affect one's psychological health and well-being, and in such a case, the impact of work on need satisfaction may be weakened (Blustein, 2006). Therefore, the PWT argues that it is necessary to improve individual satisfaction by securing decent work (Blustein et al., 2008). In the following section, we discuss the general concept of these needs and provide evidence as to how each need could affect work and the psychological well-being of individuals. In addition, we address the conceptualization of needs applied to the PWT.

#### Survival Needs

Many psychological theories consider survival needs—that is, those related to food, water, safety, and capital (Duffy et al., 2016)—as the most basic needs (Alderfer, 1969; Maslow, 1970; Glasser, 1985). Several studies have confirmed a positive relationship between satisfying survival needs and psychological well-being (Sirgy et al., 2001; Tay and Diener, 2011; e.g., Kim et al., 2017). Research in Korea has found that fulfilling one's survival needs not only enhances adaptation to school life (Park and Yang, 2015; Yang, 2015) but is also positively associated with mental health (Lim, 2008). Another study found that fulfillment of survival needs, especially economic satisfaction, had a positive effect on subjective well-being throughout all stages of life (Kim et al., 2014).

With an economic recession aggravating job instability and threatening working people's livelihoods, the need to address psychological well-being through the satisfaction of survival needs has also increased in the field of employment research (Ahn and Jung, 2019). Work has traditionally been considered as a tool to earn the income necessary for survival and has also shaped the ways in which individuals evaluate their existence (Bellah et al., 1985). Furthermore, access to the resources required for survival is regarded as a key requirement of work (Maslow, 1948; Blustein, 2006). The PWT posits that securing decent work fulfills survival needs through providing adequate rewards and safe working conditions (Duffy et al., 2016). Thus, the theory rests on the idea that work provides access to means of survival such as food, housing, and health care (Autin et al., 2019).

#### Social Contribution Needs

Human beings instinctively want to be socially connected, have healthy attachments, and feel that they belong (Bowlby, 1982; Baumeister and Leary, 1995). Work provides individuals with a pathway to fulfilling these needs (Blustein, 2006). Initial PWT research emphasized the importance of fulfilling the need for social connectedness through work, referred to as “social connection needs,” (Duffy et al., 2016) which are defined in the PWT as an individual's desire to be part of a connected community (Blustein, 2011; Duffy et al., 2016).

Since the publication of this initial research, the definition has been narrowed, since social connection needs overlap with SDT-relatedness needs (Autin et al., 2019). Social connection needs can be fulfilled directly via relationships or indirectly by contributing to greater social benefits (Blustein, 2006). Researchers have focused on the latter, which is a feeling of connectedness to broader society via increasing community welfare (Blustein, 2019). To distinguish the concepts of individual- and broader-level social connection needs, Autin et al. (2019) developed a need satisfaction scale and renamed “social connection needs” as “social contribution needs.” This change stresses the need to feel a broad social connection. Therefore, it is important to emphasize that in the context of a broader sense of connection, social contribution needs are distinct from relatedness needs, which amount to individual-level connections (Autin et al., 2019).

Previous studies have demonstrated that contributing to the social or economic well-being of others enhances individuals' overall well-being and job satisfaction (Grant and Berg, 2011). Additionally, individuals who perceive broader social connections tend to feel more positively about work, leading to greater job and life satisfaction (Duffy et al., 2011). The few studies available on this link found that the pursuit of the common good had a positive effect on job performance among Korean working adults (Kim, 2016). Moreover, corporate employees consider their work to be more meaningful and experience greater organizational identification when they feel that their company promotes corporate social responsibility (Kim et al., 2015). Thus, we predict that fulfilling social contribution needs will have a positive effect on job performance and work satisfaction among Korean adults.

#### Self-Determination Needs

Self-determination needs (SDNs) are basic psychological needs that are innate, “psychological nutriments” necessary for psychological growth, coherent functioning, and well-being in the SDT (Deci and Ryan, 2000, 2008). The SDT is a meta-theory that details how extrinsic motivational factors are internalized, while exploring social and environmental variables that support or hamper psychological development (Kim H. J., 2010). Basic psychological needs include autonomy, relatedness, and competence. According to the basic needs perspective, needs are universal and appear at all developmental stages in every culture (Ryan and Deci, 2002). Although previous studies have verified that relatedness needs tend to be emphasized more in collectivistic cultures compared to in individualistic cultures, the universality of needs and the benefits of satisfying those needs have been confirmed beyond the boundaries of race or culture (Chirkov et al., 2005; Kim and Lee, 2006; Lee and Kim, 2008; Oh et al., 2008; Han and Shin, 2009; Taylor and Lonsdale, 2010). These basic, inherent, and universal needs affect an individual's degree of satisfaction when interacting with the social environment (Deci and Vansteenkiste, 2004).

Several previous studies have verified the association between satisfaction of these three needs and well-being indicators (Vansteenkiste and Ryan, 2013). Meeting SDNs enhances intrinsic motivation and is positively correlated with job satisfaction (Kasser et al., 1992; Ilardi et al., 1993; Gagn et al., 2000; Deci et al., 2001; Baard et al., 2004). Such links have also been reported among Koreans; satisfying SDN has been shown to positively influence employee organizational commitment (Chang and Choi, 2007) and improve individual health as well as psychological well-being (Kim and Lee, 2008; Kwak and Son, 2008; Han and Shin, 2009; Sihn et al., 2010).

Under the SDT, autonomy is an individual's desire to act freely, with a sense of control over their own choices (Deci and Ryan, 2000). Autonomy is considered as essential for self-motivation (Sheldon et al., 2017). Relatedness is an individual's desire to feel a sense of intimacy with others in a community, caring for them, and feeling cared for by them (Baumeister and Leary, 1995). In healthy and safe working conditions, assurance of belonging generates personal connections, satisfying the need for relatedness (Autin et al., 2019). Finally, competence is an individual's desire to develop mastery and skills within a given environment. When these three needs are satisfied in work settings, workers not only feel control over given tasks, but create genuine connections with their tasks (Van den Broeck et al., 2016). Autonomy, relatedness, and competence are all needed to enhance employees' psychological growth and well-being (Van den Broeck et al., 2016).

The PWT includes autonomy, relatedness, and competence as outcomes of decent work (Duffy et al., 2016) and assumes that meeting these three SDNs through decent work increases self-determination and enables adequate functioning in real life, where most people cannot choose certain types of work from purely intrinsic motivations (Blustein, 2006). More specifically, an appropriately supplied external environment provides adequate extrinsic benefits that work with intrinsic motivation to prepare a pathway toward meaningful work. The PWT-based WNSS (Autin et al., 2019) defines the three SDNs as follows: first, autonomy is defined as a desire to live an independent life and to pursue living one's life in the form of integrated self; second, competence reflects the need to have control over one's environment and to achieve mastery; last, relatedness is a concept relating to the need to engage and care for others.

### The Korean Context

In Korea, most studies regarding need satisfaction have revolved around the three SDNs. Basic psychological needs scales have also been developed and validated under different contexts. Need satisfaction scales have been developed for couples (Han and Shin, 2006) and the parent-child relationship (Han and Shin, 2010; Jeong and Shin, 2010). In counseling settings, basic psychological need satisfaction scales have been developed for clients (Han and Shin, 2010) and counselors (Lim and Kim, 2015). Other psychological needs scales have also been generated for people with physical disabilities (Kim, 2011), for physical education classes (Park and Kim, 2008; Park, 2009), and for adolescents (Lee and Kim, 2008; Kim H. J., 2010; Jeon, 2014). However, studies related to the development of a need satisfaction scale in Korea have focused primarily on education and sports sites, and research using the themes of adults or work is insufficient (Hwang and Kim, 2013). Even the existing need satisfaction scales are largely based on the SDT and thus do not include survival needs or social contribution needs from the PWT. This despite all the needs from the PWT being vital for the achievement of psychological well-being in working adults. Therefore, a new scale to compensate for the lack of existing scales and to fully measure the satisfaction of all five dimensions of needs referred to in the WNSS is needed in Korea.

Since the 1960s, the Korean economy has experienced unprecedented rapid economic growth. Currently, Korea is the world's eleventh largest economy with its GDP exceeding $1.41 trillion as of 2018 (Nam and Kim, 2019). Nevertheless, the Korean labor market faces several challenges. The youth (15–24 years) unemployment rate is 10.4% (OECD, 2018a), 6.7% higher than the total unemployment rate (OECD, 2018b), which is accompanied by an aging society, low fertility, and economic inequality (Nam and Kim, 2019). Given this social environment, the need to apply the PWT to Korean society is increasing (Ahn and Jung, 2019; Nam and Kim, 2019).

On average, Koreans work 1,993 h annually, which is 259 h longer than the OECD's average of 1,730 h (OECD, 2018c). In response to this, the government enacted a law in July 2018 that set a limit of 52 working h per week (Nam and Kim, 2019). Additionally, there has been increasing demand to improve work–life balance among employees. According to recent research, Korean workers recognize “decent work” as work that guarantees sufficient free time and rest time. Further, they are of the view that companies should respect working-hour restrictions and legal holidays (Nam and Kim, 2019). Furthermore, the demand for physically and emotionally safe working conditions is increasing (Seo et al., 2012), as is the demand for reforms to tackle workplace bullying (Kim G. J., 2019). These perception changes regarding work in Korea (e.g., long hours and workplace relational problems) show that work has a strong influence on individuals and that Koreans are becoming increasingly aware that their working conditions should be improved. Nevertheless, to date, no research has examined work from the perspective of need satisfaction, and therefore, this study aimed to analyze Korean workers from the need satisfaction point of view.

Specifically, the goal of this study was to adapt and validate the WNSS (Autin et al., 2019) for Korean workers (the K-WNSS). We intended to determine the factor structure of the K-WNSS through exploratory factor analysis (EFA). We then used confirmatory factor analysis to identify the competing model, and finally tested the convergent, discriminant, and incremental validity of the K-WNSS.

## Stage 1: Development of the Korean Version of the Work Need Satisfaction Scale for Working Adults

### Materials and Methods

#### Development of the WNSS Scale

The 20 items comprising the WNSS for working adults (Autin et al., 2019) assess five distinct dimensions of needs considered to be satisfied through decent work (i.e., five subscales with four items each). Each item is rated on a 7-point Likert scale, ranging from 1 = *strongly disagree* to 7 = *strongly agree*. Forward adaptation and back-adaptation are two techniques typically used to adjust scales in cross-cultural studies. Here, we chose back-adaptation because it is the most efficient method of securing inter-testing equivalence when adopting a foreign scale (Kim and Lim, 2003). First, the principal researcher and two co-researchers translated the original scale into Korean. We focused on using Korean expressions that conveyed the appropriate meaning rather than literal translations. For example, the original scale began with “My work allows me to,” but this was difficult to express in Korean. Instead, we added the following instructional sentence: “Please check the appropriate box to indicate how much your current work satisfies each of the following needs.” The draft adaptation was back-translated by a bilingual psychology professor and a Ph.D. student majoring in psychology, both living in the United States. The back-translation was sent to the developer of the original scale for similarity assessment. After modifications were made based on the feedback received, the final version of the K-WNSS was produced.

#### Participants

The participants were 589 working adults (≥19 years) in South Korea. Of these participants, 339 were included in the EFA. The mean age was 38.85 years (SD = 7.66), with a range of 27–57 years. Of the total, 57.2% (*n* = 194) identified themselves as men and 42.8% (*n* = 145) as women. In terms of education, 0.9% (*n* = 3) completed middle school or elementary school, 11.5% (*n* = 39) finished high school, 15.3% (*n* = 52) finished junior college, 23.9% (*n* = 81) graduated from Seoul-based universities, 36.6% (*n* = 124) graduated from provincial universities, and 11.8% (*n* = 40) had postgraduate education. The distributions of regular (permanent) and temporary employment were 91.4% (*n* = 310) and 8.6% (*n* = 29), respectively. Employees were 67.8% (*n* = 230) of the total sample; professionals were 13.35% (*n* = 45); freelancers were 7.1% (*n* = 14); self-employed were 7.1% (*n* = 24); and others were 7.7% (*n* = 26). Within their workplaces, 33.0% (*n* = 112) were employees/assistant team leaders; 35.1% (*n* = 119) were team leaders/section chiefs; 21.2% (*n* = 72) were deputy general managers/general managers; and 10.6% (*n* = 35) were others. Annual salaries were <20 million won for 10.6% (*n* = 36), 20–40 million won for 47.8% (*n* = 162), 40–60 million won for 22.1% (*n* = 75), 60–80 million won for 11.8% (*n* = 40), 80–100 million won for 5.0% (*n* = 17), and > 100 million won for 2.7% (*n* = 9). Self-reported social status was determined on a scale of 1 (worst off) to 10 (best off): 18.0% (*n* = 61) chose 1–3; 75.8% (*n* = 257) chose 4–7; and 6.2% (*n* = 21) chose 8–10.

#### Procedures

This study was approved by the Institutional Review Board of Yonsei University. Online questionnaires consisting of demographic questions and psychometric scales were administered to working adults (≥19 years) through the online data collection company, Data Spring. The survey consisted of 20 items from the K-WNSS, and items on demographic information—age, gender, employment status, etc.—along with six variables (decent work, job satisfaction, life satisfaction, Maslow's needs, work as meaning, and basic psychological needs) to check validity in the second stage. Workers who registered as research participants on the online survey company's website and wished to participate in the study were informed of the study's purpose. They were also informed that their participation was voluntary, that they could withdraw their participation at any time, and that no identifiable information would be collected. Participants who completed the survey were rewarded with 670 points on the survey site. The survey took ~10 min to complete.

#### Measures

##### Work Need Satisfaction

The WNSS (Autin et al., 2019) was adapted and used for validating the K-WNSS. The WNSS measures the degree of need satisfaction in three primary domains that can be fulfilled through decent work. It consists of 20 items evenly separated into five subscales: Survival, Social Contribution, Competence, Relatedness, and Autonomy. Each item is rated on a 7-point Likert scale (1 = *strongly disagree*, 7 = *strongly agree*). Example items are: “I can pay for adequate housing for my family,” “I feel like I am good at my job,” and “I feel like I fit in.” The range of Cronbach's α for the subscales was 0.85–0.95 in Autin et al. (2019).

##### Decent Work

The DWS (Duffy et al., 2017) was adapted and validated by Nam and Kim (2019). This study used the validated Korean version (K-DWS), consisting of 15 items equally distributed across five subscales: Safe working conditions, Access to healthcare, Adequate compensation, Free time/rest, and Complementary value. Each item is rated on a 7-point Likert scale (1 = *strongly disagree*, 7 = *strongly agree*). Example items are: “I feel emotionally safe interacting with people at work,” “I am not properly paid for my work (plus the reverse),” and “I can have free time during working days.” Ranges of Cronbach's α for the subscales were 0.79–0.95 in Duffy et al. (2017), 0.79–0.97 in Nam and Kim (2019), and 0.74–0.94 in this study. McDonald's ω was 0.85 [0.80, 0.89] in this study.

##### Job Satisfaction

Job satisfaction was assessed using the Job Satisfaction Scale (JSS; Judge et al., 1998), based on the job satisfaction index (Brayfield and Rothe, 1951). The first author adapted the JSS for Korean populations; bilingual experts then performed back-translation and similarity assessments. The JSS consists of five items rated on a 6-point Likert scale (1 = *strongly disagree*, 6 = *strongly agree*), including items such as: “I feel fairly satisfied with my present job” and “I consider my job rather unpleasant (plus the reverse).” Cronbach's α for this scale was 0.88 in Judge et al. (1998), 0.94 in Nam and Kim (2019), and 0.86 in this study. McDonald's ω was 0.87 [0.84, 0.90] in this study.

##### Life Satisfaction

This variable was measured using the Korean version of the Satisfaction with Life Scale (K-SWLS; Cho and Cha, 1998), adapted from the original SWLS (Diener et al., 1985) and validated by Lim (2012). The scale has five items rated on a 7-point Likert scale (1 = *strongly disagree*, 7 = *strongly agree*). Example items include: “I am satisfied with my life” and “So far I have gotten the important things I want in my life.” Cronbach's α was 0.87 in Diener et al. (1985), 0.84–0.91 in Lim (2012), and 0.93 in this study. McDonald's ω was 0.94 [0.92, 0.95] in this study.

##### Maslow's Safety–Security Needs

These variables were measured using the first author's adaptation of the “safety–security needs” subscale from Maslow's Five Needs Scale (Taormina and Gao, 2013). Bilingual experts back-translated and performed similarity assessments of the adaptation. The scale has 15 items rated on a 5-point Likert scale (1 = *strongly disagree*, 5 = *strongly agree*) including: “I am completely satisfied with the quality of the house/apartment I am living in” and “I am completely satisfied with how safe I am from catching any disease.” Cronbach's α was 0.87 in Taormina and Gao (2013), 0.90 in Autin et al. (2019), and 0.92 in this study. McDonald's ω was 0.93 [0.90, 0.94] in this study.

##### Work as Meaning

This variable was measured using the “greater social good” subscale of the Korean version of the Work as Meaning Inventory (K-WAMI), adapted and validated (Kim, 2014) from the original WAMI (Steger et al., 2012). The scale has three items rated on a 5-point Likert scale (1 = *strongly disagree*, 5 = *strongly agree*), including “I know my work makes a positive difference in the world.” Cronbach's α was 0.83 in Steger et al., 2012, 0.77 in Kim (2014), and 0.79 in this study. McDonald's ω was 0.81 [0.76, 0.86] in this study.

##### Basic Psychological Needs

These variables were measured using the Basic Psychological Needs Scale (BPNS; Lee and Kim, 2008), based on the SDT (Ryan and Deci, 2002). Currently, there is no scale in Korea for measuring needs in workplace settings. We therefore chose the K-BPNS as a replacement and added the phrase “in the workplace” to the scale's instructions. The scale has 18 items evenly divided across three subscales: Autonomy, Competence, and Relatedness. Each item is rated on a 6-point Likert scale (1 = *strongly disagree*, 6 = *strongly agree*). Examples of items are: “I feel like I am controlled and oppressed by others (plus the reverse),” “I feel like I have the ability to solve the tasks assigned to me,” and “I get along well with people I encounter.” The range of Cronbach's α was 0.70–0.79 in Lee and Kim (2008) and 0.79–0.87 in this study. McDonald's ω was 0.90 [0.89, 0.92] in this study.

### Results

#### Preliminary Analysis

Prior to analysis, skewness and kurtosis were assessed using IBM SPSS 25. Skewness and kurtosis of each item ranged from −0.52 to −1.15 and from −0.27 to 1.30, respectively, meeting normality assumptions (skewness ≤ | 2 | and kurtosis ≤ | 2 |; Garson, 2012).

#### Exploratory Factor Analysis and Reliability

To determine K-WNSS factor structure, we performed an EFA using Mplus 7.4. For EFA, the correlation matrix was used and analyzed using the maximum likelihood estimation method with goemin rotation (Muthén and Muthén, 1998–2015). The results of sample adequacy tests for factor analysis revealed a Kaiser-Meyer-Olkin index of 0.912 and significant Bartlett's test of sphericity ($\chi_{190}^{2}$ = 4584.198, *p* < 0.000). Factors were identified by evaluating models using goodness-of-fit indices: χ^2^, Tucker–Lewis Index (TLI), Comparative Fit Index (CFI), and Root Mean Square Error of Approximation (RMSEA). In contrast to χ^2^, the latter three are less sensitive to sample size and reflect model simplicity. RMSEA values of 0.08 or lower and CFI and TLI indices of 0.90 or higher indicate good model fit (Byrne, 1998).

As a result of EFA using Mplus, the 1-factor model consisted of one sub-factor with all items. The 2-factor model consisted of one sub-factor with all self-determination needs and survival needs, except for one item. A further sub-factor had social contribution needs and one item from survival needs. The 3-factor model comprised three sub-factors: survival needs, social contribution needs, and all self-determination needs. The 4-factor model was comprised of survival needs, social contribution needs, and as sub-factors, two items from relatedness and competence and two items from relatedness and autonomy. The 5-factor model consisted of five sub-factors: survival needs, social contribution needs, competence, relatedness, and autonomy. Lastly, the 6-factor model comprised four sub-factors: survival needs, social contribution needs, competence, and autonomy. One sub-factor consisted of two items from relatedness and the other sub-factor consisted of two other items from relatedness.

Although the 6-factor model showed the best model fit (Table 1), the model could be overfit to the data. Items 15 and 16 formed one sub-factor in the 6-factor model since the sentence structures of these two items were similar when they were translated into Korean. Therefore, as the researchers suggested (e.g., Cudeck and Henly, 1991; Preacher et al., 2013), we selected the 5-factor model as the competing model, which is congruent with the goal of this study and the PWT framework when considering generalizability. EFA identified the 5-factor model as having good model fit. The model fit was $\chi_{\left(100\right)}^{2}$ = 255.63, *p* < 0.000; RMSEA = 0.068 [0.06, 0.08], CFI = 0.97, TLI = 0.93. We verified that the 5-factor structure was the same as in the original scale. Additionally, all items clustered on the factors as predicted by the PWT, and the range of factor loadings for all items was 0.52–0.89 (Table 2), exceeding the cutoff value of 0.50 (Costello and Osborne, 2005). Correlations between subfactors are presented in Table 3.

**Table 1:** Exploratory factor analysis of K-WNSS *(N* = *339)*.

**Model**	**x^**2**^**	**df**	**RMSEA**	**CFI**	**TLI**
1-Factor model	1820.14	170	0.17	0.63	0.59
2-Factor model	1239.33	151	0.15	0.76	0.70
3-Factor model	914.48	133	0.13	0.83	0.75
4-Factor model	542.18	116	0.10	0.91	0.85
5-Factor model	255.63	100	0.07	0.97	0.93
6-Factor model	138.67	85	0.04	0.99	0.97

**Table 2:** Factor loadings of exploratory factor analysis (*N* = *339*).

**Items**		**Factor loadings**				
**Original**	**Korean**	**1**	**2**	**3**	**4**	**5**
My work allows me to…						
**Factor 1. Survival needs**						
have the resources to provide nutritious food for myself and my family.	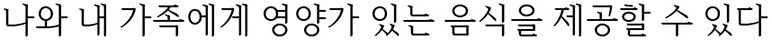	**0.575** (0.06)	0.035	0.012	0.127	−0.071
have the resources to pay for adequate housing for my family.	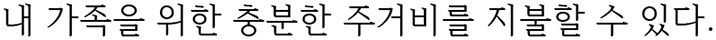	**0.867** (0.05)	0.025	−0.138	0.043	0.021
have the resources to pay for utilities, such as water, heating, and electric, on time	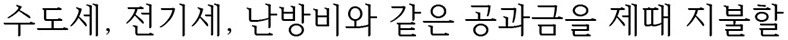 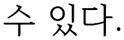	**0.614** (0.06)	−0.053	0.055	0.002	0.050
have the resources to maintain the health of myself and my family	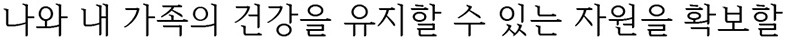 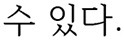	**0.815** (0.05)	0.025	0.070	−0.030	0.033
**Factor 2. Social contribution needs**						
make a contribution to the greater social good.	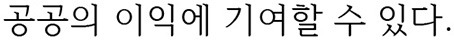	0.205	**0.671** (0.04)	0.033	−0.025	−0.003
feel like I am doing something important for my community.	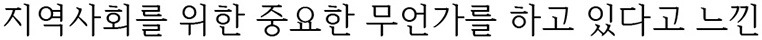 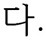	0.048	**0.853** (0.04)	−0.025	−0.055	0.004
feel a part of something greater by helping to sustain our world.	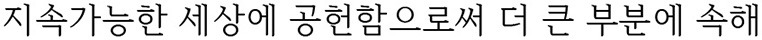 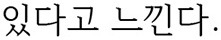	−0.053	**0.888** (0.03)	−0.011	0.054	0.055
feel like I am making a difference.	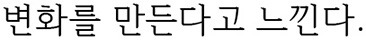	0.008	**0.555** (0.05)	0.161	0.146	−0.044
**Factor 3. Competence needs**						
feel like I am good at my job.	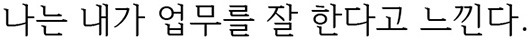	−0.008	0.027	**0.894** (0.03)	0.031	−0.016
feel like I am good at what I do.	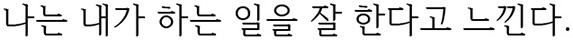	0.021	−0.003	**0.933** (0.03)	−0.046	0.034
feel like I know what I'm doing.	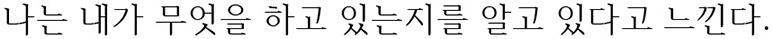	0.194	0.045	**0.538** (0.05)	0.031	0.069
feel competent.	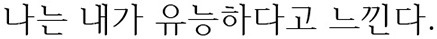	−0.045	0.021	**0.682** (0.05)	0.103	0.088
**Factor 4. Relatedness needs**						
feel like I fit in.	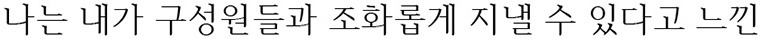 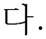	0.065	−0.098	0.272	**0.548** (0.04)	−0.019
feel like I belong.	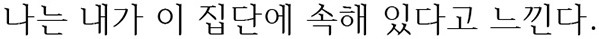	0.093	−0.007	0.188	**0.621** (0.04)	−0.028
feel understood by others.	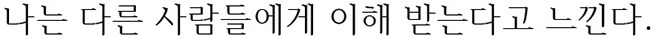	0.017	0.054	−0.079	**0.830** (0.05)	0.076
feel supported by others.	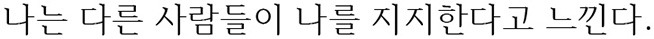	−0.095	0.020	0.025	**0.770** (0.06)	0.191
**Factor 5. Autonomy needs**						
do tasks the way I want.	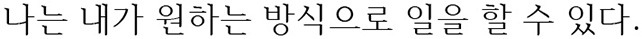	−0.006	−0.011	−0.004	0.013	**0.882** (0.04)
feel free to do things my own way.	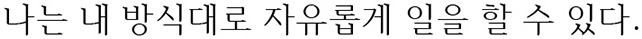	−0.015	−0.001	−0.011	−0.003	**0.894** (0.04)
take actions that promote my real needs.	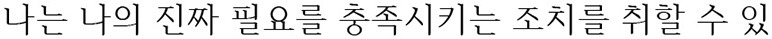 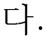	0.064	0.149	0.043	0.201	**0.522** (0.05)
choose whether or not I have to do certain tasks.	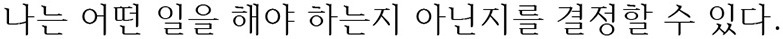	0.107	−0.012	0.132	0.005	**0.599** (0.06)

*Factor loadings > 0.50 are in boldface. Parentheses are standard errors of factor loadings. Maximum likelihood method was used*.

**Table 3:** Correlations between the K–WNSS subscales and descriptive information (*N* = *339*).

**Subscale**	**1**	**2**	**3**	**4**	**5**
1. K-WNSS-survival	1				
2. K-WNSS-social contribution	0.49	1			
3. K-WNSS-competence	0.48	0.42	1		
4. K-WNSS-relatedness	0.48	0.45	0.65	1	
5. K-WNSS-autonomy	0.45	0.44	0.64	0.64	1
*M*	5.37	4.17	5.07	4.84	4.80
*SD*	1.03	1.15	0.99	0.98	1.08
*SE*	0.06	0.06	0.05	0.05	0.06

*All correlations are significant at p <0.01. Maximum likelihood method was used*.

The coefficient omega was used to identify the reliability of the 5-factor model derived from EFA. Researchers have found that the coefficient omega is a more sensible index of reliability compared to other alternatives, including Cronbach's α (e.g., Zinbarg et al., 2006, 2007; Dunn et al., 2014). McDonald's ω was 0.93 with a 95% confidence interval from 0.91 to 0.94. We verified that the 5-factor model had good internal consistency.

## Stage 2: Confirmatory Factor Analyses and Validating the Korean Version of the Work Need Satisfaction Scale for Working Adults

### Materials and Methods

#### Participants

Data from the remaining 250 respondents were used for confirmatory factor analysis. The mean age of the respondents was 40.18 years (SD = 8.31, range = 27–65 years), including 108 men (43.2%) and 142 women (56.8%). Only two individuals (0.8%) had an education level lower than high school; 36 were high school graduates (14.4%); 36 were junior college graduates (14.4%); 69 were Seoul-based university graduates (27.6%); 84 were provincial university graduates (33.6%); and 23 completed postgraduate education (9.2%). They were mostly regular employees (221, 88.4%), with only 29 temporary employees (11.6%). Most were also employees (169, 67.6%) as opposed to professionals (30, 12.0%), freelancers (16, 6.4%), self-employed (14, 5.6%), or other (21, 8.4%). In their workplaces, 84 were employees/assistant team leaders (33.6%); 88 were team leaders/section chiefs (35.2%); 38 were deputy general managers/general managers (15.2%); and 40 were others (16.0%). The breakdown of the group by annual salary showed that 40 people (16.0%) earned <20 million won; 114 (45.6%) earned 20–40 million won; 61 (24.4%) earned 40–60 million won; 22 (8.8%) earned 60–80 million won; 7 (2.8%) earned 80–100 million won; and 6 (2.4%) earned > 100 million won. Finally, 47 (18.8%) individuals rated their social status as 1–3, 188 (75.2%) as 4–7, and 15 (6.0%) as 8–10.

### Results

#### Preliminary Analysis

Skewness and kurtosis were assessed using IBM SPSS 25. All items of each variable ranged from −0.41 to −0.08 (skewness) and −0.63 to 0.13 (kurtosis), satisfying the normality assumptions (skewness ≤ | 2 | and kurtosis ≤ | 2 |; Garson, 2012).

#### Confirmatory Factor Analysis

We conducted confirmatory factor analysis using Mplus with maximum likelihood estimation. The goodness-of-fit for the 5-factor 20-item model derived from the EFA was tested. We applied the same goodness-of-fit indices and cutoff values (Byrne, 1998) as those used in the EFA.

We compared the three models—(a) correlational model, (b) higher-order model, and (c) higher-order SDN model—in Mplus to identify the best fit for the Korean samples. Each model was selected to validate whether each five needs work distinctively rather than loading onto a higher order factor. And higher-order SDN model was compared to validate three self-determination needs are structured within the PWT as PWT framework supposed. As χ^2^ is highly sensitive to sample size, we also used CFI and RMSEA to verify significant model-fit differences, as suggested by many researchers (e.g., Cheung and Rensvold, 2002; Chen, 2007). Models were determined as invariant when ΔCFI <0.01 and ΔRMSEA <0.015 (Cheung and Rensvold, 2002; Chen, 2007). Akaike Information Criterion (AIC) and Bayes Information Criterion (BIC; Tabachnick et al., 2007) were used concurrently to compare across non-nested models. Although there are no significant cutoff criteria, the models are considered a good fit with low AIC and BIC values (Maslowsky et al., 2015).

**Figure 1:**
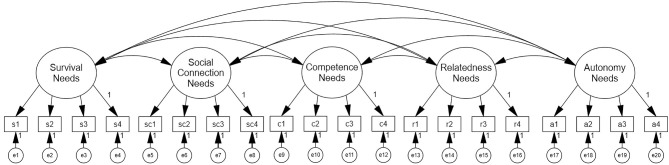
Correlational model.

The correlational model (Figure 1) has five distinct but related latent indicators, rather than one higher-order factor. Its model fit was $\chi_{160}^{2}$ = 355.219, *p* < 0.000; RMSEA = 0.070 [0.06, 0.08], CFI = 0.95, TLI = 0.94, AIC = 13177.657, and BIC = 13424.159.

**Figure 2:**
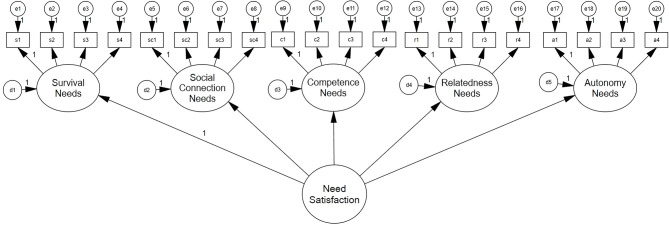
Higher-order model.

The higher-order model (Figure 2) has one higher-order construct (“need satisfaction”) with five indicators (survival, social connection, competence, relatedness, and autonomy). Factor loadings for the higher-order need satisfaction construct were as follows: Survival (*r* = 0.72), Social contribution (*r* = 0.73), Autonomy (*r* = 0.78), Competence (*r* = 0.89), Relatedness (*r* = 0.82). Model fit was $\chi_{165}^{2}$ = 371.552, *p* < 0.000; RMSEA = 0.071 [.06, 0.08], CFI = 0.94, TLI = 0.94, AIC = 13183.991, and BIC = 13412.885. There was minimal difference between the higher-order model and the correlational model (ΔCFI <0.01, ΔRMSEA <0.015, ΔAIC = −6.33, ΔBIC = 11.27).

**Figure 3:**
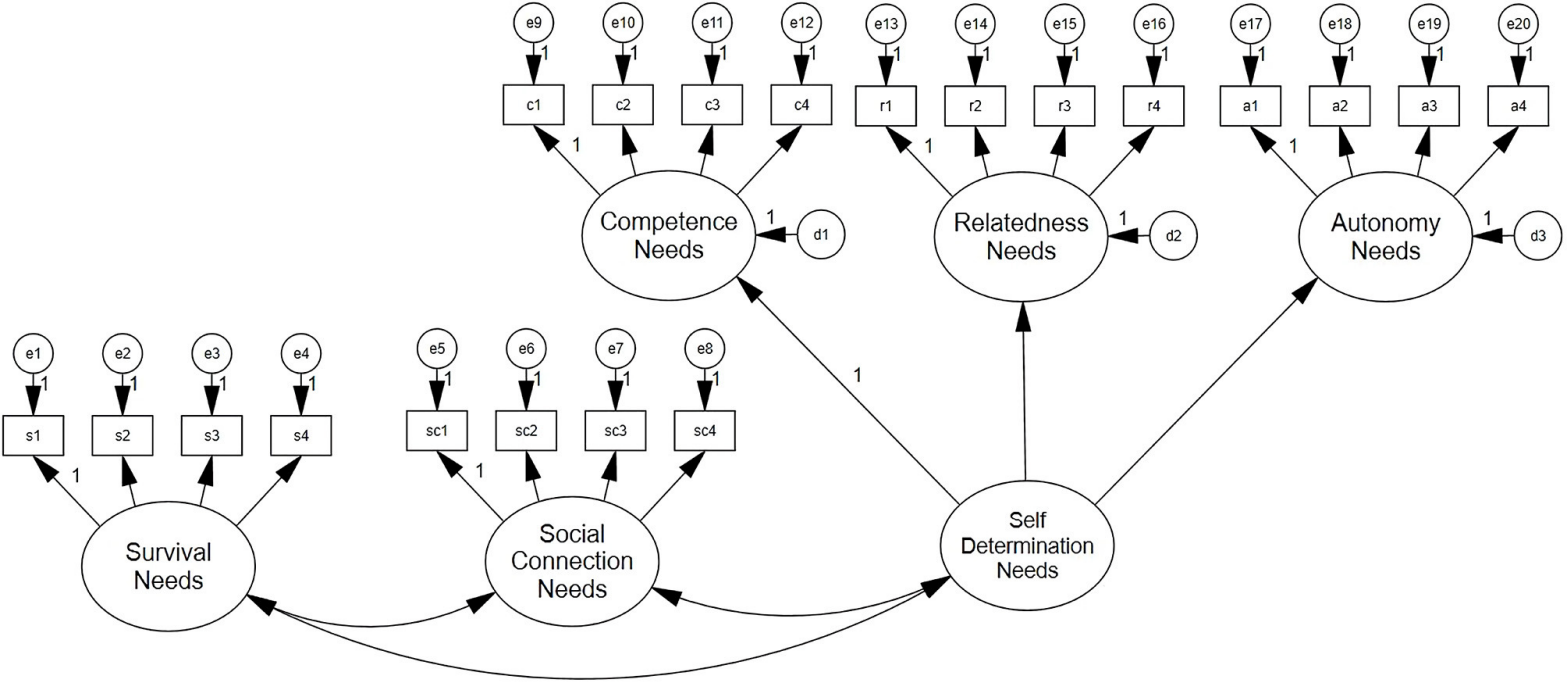
Higher-order SDN model.

The higher-order SDN model (Figure 3) has a higher-order construct (“SDN”) with three indicators (competence, relatedness, and autonomy); this construct is correlated with the remaining survival and social contribution needs. Factor loadings for the SDN construct were as follows: Autonomy (*r* = 0.79), Competence (*r* = 0.90), and Relatedness (*r* = 0.82). Model fit was $\chi_{164}^{2}$ = 361.766, *p* < 0.000; RMSEA = 0.069 [0.06, 0.08], CFI = 0.95, and TLI = 0.94. The SDN model had the lowest AIC (13176.205) and BIC (13408.621) across the three models, and it was not significantly different from the correlational model (ΔCFI <0.01, ΔRMSEA <0.015, ΔAIC = 1.45, ΔBIC = 15.54) or the higher-order model (ΔCFI <0.01, ΔRMSEA <0.015, ΔAIC = 7.79, ΔBIC = 4.26), which were similar to the original scale (Autin et al., 2019).

Although the higher-order SDN model yielded the lowest AIC and BIC, there were no significant differences in all other model fit indices with very minimal gaps. PWT supposed that the SDN is structured in the PWT framework as psychological needs and thus, we selected the higher-order SDN model for use with Korean samples, in line with the PWT (Duffy et al., 2016) and SDT (Ryan and Deci, 2017).

The coefficient omega was used to identify the reliability of the higher-order SDN model. McDonald's ω was tested and found to be 0.94 with a 95% confidence interval from 0.93 to 0.95. The results showed that the higher-order SDN model has good internal consistency.

#### Convergent and Discriminant Validity

Convergent validity is established when test scores representing a certain construct measured by different scales are more closely related to each other than different constructs measured by the same, or different scales (Baumgarten and Wetzel, 2017). Thus, we compared the relative strength of correlation coefficients between all related variables and those between all unrelated variables. We used Pearson's correlational analyses to test the associations between each of the five work needs and constructs identified as conceptually related to the five work needs, namely Maslow's safety–security needs, and self-determination needs measured by the K-BPNS. Autin et al. (2019) also included prosocial intentions for social contribution needs; however, since a suitable scale to measure this variable does not exist for Koreans, we used the “greater social good” subscale of the K-WAMI, which captures characteristics similar to the WNSS social contribution needs. We observed that related variables were convergent to each other when correlations of related variables were greater than those of unrelated ones. Except in the case of autonomy, related variables had higher correlations and were identified as convergent (Table 4). Additionally, as shown in Table 4, survival needs and Maslow's safety–security needs were correlated (*r* = 0.48), as were social contribution needs and greater social good (*r* = 0.56). All three SDN sub-factors measured using the K-WNSS were correlated with the three SDNs measured by the K-BPNS (*r* = 0.72 for competence, *r* = 0.64 for relatedness, and *r* = 0.51 for autonomy). A correlation coefficient above 0.5 is regarded as a reasonable establishment of convergent validity (Carlson and Herdman, 2012), and we observed that all related variables were convergent except for survival and Maslow's safety–security needs. However, as this correlation was significant at level *p* < 0.05, and greater than that of unrelated ones, we observed that survival and Maslow's needs were marginally convergent in this study.

**Table 4:** Descriptive data and bivariate correlations for variables used (*N* = *250*).

	**1**	**2**	**3**	**4**	**5**	**6**	**7**	**8**	**9**	**10**	**11**	**12**	**13**
1. K-WNSS-survival	1												
2. K-WNSS-social contribution	0.56	1											
3. K-WNSS-competence	0.47	0.49	1										
4. K-WNSS-relatedness	0.55	0.60	0.68	1									
5. K-WNSS-autonomy	0.51	0.57	0.64	0.66	1								
6. Decent work	0.52	0.55	0.45	0.59	0.59	1							
7. Job satisfaction	0.32	0.46	0.38	0.48	0.46	0.70	1						
8. Life satisfaction	0.40	0.53	0.40	0.55	0.52	0.62	0.57	1					
9. Maslow needs	0.48	0.45	0.37	0.52	0.45	0.58	0.46	0.71	1				
10. WAMI-greater social good	0.30	0.56	0.42	0.51	0.45	0.46	0.46	0.56	0.47	1			
11. SDT-competence	0.36	0.45	0.72	0.63	0.61	0.48	0.45	0.53	0.38	0.50	1		
12. SDT-relatedness	0.30	0.40	0.48	0.64	0.48	0.42	0.33	0.39	0.40	0.38	0.60	1	
13. SDT-autonomy	0.31	0.40	0.43	0.45	0.51	0.45	0.47	0.37	0.31	0.46	0.48	0.49	1
*M*	5.31	4.14	5.09	4.87	4.69	4.29	3.12	3.67	3.18	3.18	4.16	4.41	4.00
*SD*	1.14	1.19	1.02	1.04	1.16	0.90	0.77	1.45	0.80	0.80	0.80	0.78	0.79
*SE*	0.07	0.07	0.06	0.07	0.07	0.06	0.05	0.09	0.04	0.05	0.05	0.05	0.05

*All correlations are significant at the p <0.01 level*.

To establish the discriminant validity, we assessed the heterotrait-monotrait ratio of correlations (HTMT) of each related convergent variable. HTMT is an effective method to investigate similarity of latent variables (Henseler et al., 2015). A HTMT value <1 is regarded as establishment of discriminant validity and some researchers suggest that the conservative standard is 0.85 (Clark and Watson, 1995; Kline, 2011). As a result, HTMT values were 0.53 for survival needs and Maslow's safety–security needs, 0.46 for social contribution needs and prosocial intentions, 0.61 for autonomy, 0.81 for competence, and 0.73 for relatedness measured by each K-BPNS and K-WNSS. We observed that all related convergent variables were discriminant as well, since all HTMT values were under 0.85.

#### Concurrent Validity

Concurrent validity was tested to identify whether each of the five needs measured by the K-WNSS uniquely predicted the work fulfillment and well-being that the PWT model proposes. A relative weight analysis (RWA; Johnson, 2000), also referred to as relative importance analysis, was performed using RWA-Web (Tonidandel and LeBreton, 2014). This method aims to identify each predictor's unique contribution to a criterion and the proportion of explained variance each predictor accounts for in the total criteria variance. RWA is useful when predictors are correlated with one another and result in multicollinearity issues. The significance of individual relative weight was tested using 95% confidence intervals (Tonidandel et al., 2009).

Results from the RWA are presented in Table 5. Job satisfaction is an indicator of work fulfillment and life satisfaction is an indicator of well-being. The results indicated that the five needs explained roughly 30% of the variance in job satisfaction (*R*^2^ = 0.28), and 40% in life satisfaction (*R*^2^ = 0.39). An examination of the RWA revealed that all five needs explained a statistically significant amount of variance in both job satisfaction and life satisfaction as all confidence intervals were not included at zero. Specifically, relatedness explained the largest proportion of the variance in job satisfaction (RW = 0.08), followed by social contribution (RW = 0.07), autonomy (RW = 0.07), and survival (RW = 0.02). Social contribution was the most important need for explaining the variance in life satisfaction (RW = 0.11), followed by relatedness (RW = 0.11), autonomy (RW = 0.10), and survival (RW = 0.04).

**Table 5:** Relative weight analysis for concurrent validity (*N* = *250*).

	**Job satisfaction (*****R*******^****2****^ **=** **0.28)**				**Life satisfaction (*****R*******^****2****^ **=** **0.39)**			
	**RW**	**% of *R*^2^**	**CI-L**	**CI-U**	**RW**	**% of *R*^2^**	**CI-L**	**CI-U**
Survival	0.02	8.5%	0.001	0.05	0.04	10.8%	0.01	0.08
Social contribution	0.07	25.8%	0.03	0.13	0.11	28.3%	0.06	0.17
Competence	0.04	13.3%	0.01	0.08	0.04	9.8%	0.01	0.07
Relatedness	0.08	27.4%	0.04	0.13	0.11	27.5%	0.06	0.16
Autonomy	0.07	25.0%	0.02	0.13	0.10	23.6%	0.05	0.14

*All confidence intervals are significant at the p <0.05 level. RW, Raw Weight; CI-L, Confidence Interval-Lower Bound; CI-U, Confidence Interval-Upper Bound*.

## Discussion

This study aimed to deliver a reliable scale to measure need satisfaction in work settings for Korean working adults. To achieve this, we translated the original scale and then tested the validity and reliability of the translated scale. Five factors were extracted and confirmed to verify the theoretical assumptions of the work need satisfaction construct in the subsequent process of securing decent work based on PWT (Duffy et al., 2016). All factors were found to be positively related and could also be differentiated. We noted good internal consistency for the 5-factor model derived from EFA using McDonald's ω. Additionally, we found that the correlational, higher-order, and higher-order SDN models were all a good fit for the Korean data, with few inter-model differences. Consequently, we selected the higher-order SDN model for the Korean data as identically validated by the WNSS development study (Autin et al., 2019). The reasons for the selection are as follows: First, although the three models are equivalent to each other in terms of model fit, the higher-order model has relatively better fit out of all three models. Moreover, having a higher-order factor is found to be more parsimonious than having five factors. Second, the higher-order SND model is in accordance with the PWT framework in which psychological needs fulfillment is considered as a composite experience. However, depending on varying study objectives, such as verifying the uniqueness of SDT needs, the correlational model with identical model fit can be used for Korean data.

The K-WNSS demonstrated good convergent and discriminant validity through correlations with related and unrelated constructs. Working adults whose needs are satisfied by their work generally report higher fulfillment in both their jobs and overall lives. Moreover, those who regarded their work as decent reported greater satisfaction of needs from work. In particular, we noted that the autonomy of the K-WNSS was the most highly correlated with competence from the K-BPNS and had the second highest correlation with autonomy from the K-BPNS. This outcome is consistent with previous studies conducted in Korea showing that the three basic psychological needs are closely interrelated (e.g., Han and Shin, 2009; Jeong and Shin, 2010), especially in terms of autonomy and competence (Hwang and Kim, 2013). According to the SDT, autonomy is the most important of the three basic needs, given that competence depends on the assurance of autonomy (Ryan, 1982). However, some Korean studies indicated that competence has a greater effect on intrinsic motivation and achievement than autonomy (Lee and Jung, 2008; Hwang and Kim, 2013; Kim and Yoon, 2013). The nation has undergone rapid economic growth since the Korean War, and Korean companies have become some of the leading global corporations. This growth rate was possible because Korean society generated an organizational culture capable of yielding rapid results, fostering a corporate culture with deeply rooted hierarchical rankism (Lee, 2014). Thus, in the organizational culture of Korea, the autonomy that one holds when making decisions reflects one's ability to stand out with competence. The interconnectedness of autonomy and competence led Jeong and Shin (2010) to propose that the three basic psychological needs of SDT are closely interconnected. We therefore concluded that we had established convergent validity, as we found significant correlations between autonomy, competence, and relatedness from our scale and the existing scales.

We demonstrated that the K-WNSS has concurrent validity, and all five factors statistically explain the variance of criteria, which are job satisfaction and life satisfaction as proposed by the PWT. The results revealed that relatedness is the most important need to explain job satisfaction. It may be that relatedness is the more salient need in one's work fulfillment in Korea. Previous studies conducted in Korea have confirmed that relatedness needs tend to be more valued and stressed in a collectivistic culture (Kim and Lee, 2006; Lee and Kim, 2008; Oh et al., 2008). Several studies (in Korea and abroad) showed that good quality of interpersonal relationships at work had significant positive effects on job satisfaction and commitment, whereas interpersonal conflict at work had a negative effect on various organizational outcomes (e.g., Simons and Peterson, 2000; Jehn and Mannix, 2001; Hwang, 2007; Lee and Park, 2011). Additionally, social contribution needs explained the largest proportion of variance in life satisfaction. This shows that one's perception of how meaningful one's work is and how much it is contributing to society, might be more crucial for engendering well-being among Korean workers. This result is supported by the research that found that perceiving one's work as meaningful and that it was a calling was linked to lifetime achievements such as life satisfaction (e.g., Steger et al., 2010; Lips-Wiersma and Wright, 2012; Jang and Lee, 2014). This validity finding confirms that all sub-factors of the K-WNSS are essential for explaining working adults' experiences of satisfaction at their work and in their lives in Korea. Further, this theoretically supports the PWT by demonstrating that survival and social contribution needs should be included into needs through work. The results also identify the K-WNSS as a useful scale that predicts job and life satisfaction in Korea.

As expected, the structure of the K-WNSS was remarkably similar to that of the original scale (Autin et al., 2019), with the exception of the high correlation between competence and autonomy as mentioned above. No significant differences were observed at either the structural or factorial level. These results indicate that needs are universal in every culture, which is true even in work settings. In previous studies, basic psychological need satisfaction predicted personal well-being in various cultures worldwide (Chirkov et al., 2003, 2005; Han and Shin, 2009; Sihn et al., 2010; Taylor and Lonsdale, 2010). Chen et al. (2015) validated that the positive relation between need satisfaction and well-being was found in samples in Peru, Belgium, the US, and China, and that this relation was not moderated by cultural backgrounds. Overall, we offer a cross-culturally valid and reliable tool for Korean working adults to measure the degree to which work, in pursuit of work fulfillment and well-being, satisfies basic but core needs: to survive, to contribute socially, to relate to others, and to be autonomous and competitive at work.

### Implications

Our primary contribution is adapting and validating the WNSS for working Koreans. Prior to this study, no such scale was available to measure fulfillment of needs through work in Korea. To date, few studies have investigated working adults, companies, and jobs in the context of needs fulfillment (Hwang and Kim, 2013). Therefore, this study can be an important starting point for future research on the determinants and effects of needs fulfillment in work settings, where adults usually spend most of their time. This study is also significant in that it successfully applied the PWT to a rapidly changing domestic labor market, allowing empirical study of the theory's entire pathway. In particular, the survival need satisfaction subscale may be useful for marginalized populations as it is closely related to power and sociocultural privilege. In Korea, income inequality is relatively high (OECD, 2021), and issues of underemployment are severe (Kim and Allan, 2020). In addition, marginalized individuals are vulnerable to structural and environmental changes such as the COVID-19 pandemic (Blustein et al., 2020). By using the subscale, we will be able to assess the degree of survival need satisfaction and find out its effects on the work and life domain satisfaction of marginalized individuals.

Previous applications of PWT in Korea directly predicted job satisfaction, but not life satisfaction (Lee and Lee, 2020). Elsewhere, Duffy et al. (2019) reported that decent work had a direct, significant effect on the physical health of American workers, but not on their mental health. However, a significant pathway has been identified when work needs fulfillment mediates these two outcomes. This finding suggests that needs fulfillment plays a major mediating role between decent work (predictor) and outcomes, as assumed by the PWT. Our application of the PWT using the K-WNSS will enable sophisticated empirical verification of such mediating effects in Korea.

Understanding an individual's basic needs can benefit efforts to overcome needs-related barriers. With the K-WNSS, we will be able to quantify need satisfaction during ongoing work and provide employees with insight into how their employment fulfills their needs. As a key element of the human experience, work should be a primary focus in counseling settings (Richardson, 1993; Peterson and Gonzalez, 2005; Blustein et al., 2008). The K-WNSS can be a useful tool for corporate counseling and career development, allowing prompt intervention to enhance employees' job and life satisfaction.

Lastly, we also recommend national policies that consider needs fulfillment to promote worker well-being. During the COVID-19 pandemic, massive underemployment has occurred, gravely affecting individuals' well-being (Blustein, 2019; Blustein et al., 2020). In response to this reality, the need for policy guidance is increasing (Blustein et al., 2020). Therefore, we suggest that the government should make efforts to generate decent jobs that can satisfy individuals' needs on a macroscopic level, ensuring appropriate job conditions while improving the treatment of underemployed and temporary workers.

### Limitations and Future Directions

This study has several limitations. First, we still need to establish the predictive validity of the K-WNSS by examining its predictive power. Specifically, future studies should confirm whether satisfying work needs actually mediates between decent work and health outcomes, as predicted by the PWT. Such results would strengthen the reliability and validity of the K-WNSS.

Second, we require a broader range of studies on a more diverse groups of adults. The use of panel data on online data collection platforms creates concerns about the representativeness of data (Ophir et al., 2019). In addition, traditionally, Korea has been considered as a homogenous nation without many prominent racial issues that act as major discrimination factors in other countries. However, the number of foreigners currently residing in Korea has exceeded 2 million, and nearly half (990,000) are migrant workers (Korea Statistics, 2019a). In addition, over 30,000 North Korean defectors (*Saeteomin*, or new settlement people) have entered South Korea since 1998 (Korea Ministry of Unification, 2020). Accordingly, understanding cultural differences and resolving multicultural conflicts have become emerging social issues in Korea (Kim, 2008). Moreover, specific types of jobs could have different effects on work need satisfaction (i.e., white collar vs. blue collar or office workers vs. private business owners). Thus, future research on the K-WNSS must include various racial or socioeconomic samples.

Lastly, a follow-up qualitative study would be necessary to determine whether there are cultural differences in work need satisfaction. Korea has different working conditions from the US, where the WNSS was developed. There may also be differences stemming from the relationship-oriented Korean culture. For example, as shown in the finding of a qualitative analysis of basic psychological needs among Korean adolescents, the notion of competence as perceived by Korean adolescents includes a sense of superiority when comparing themselves with others (Lee and Kim, 2008). We can gain meaningful insight via a study that determines the needs Koreans want to fulfill through work, and then conduct cross-cultural comparisons.

## Data Availability Statement

The datasets generated for this study are available on request to the corresponding author.

## Ethics Statement

The studies involving human participants were reviewed and approved by Yonsei University Institutional Review Board. Written informed consent for participation was not required for this study in accordance with the institutional requirements.

## Author Contributions

J-HK designed the study, collected the data, and performed the data analysis under the supervision of K-HL. All authors provided ideas, contributed to writing the article, and approved the final version of the manuscript for submission.

## Conflict of Interest

The authors declare that the research was conducted in the absence of any commercial or financial relationships that could be construed as a potential conflict of interest.
